# Examining the Impact of a Personalized Self-Management Lifestyle Program Using Mobile Technology on the Health and Well-Being of Cancer Survivors: Protocol and Rationale for a Randomized Controlled Trial (The Moving On Study)

**DOI:** 10.2196/13214

**Published:** 2019-08-23

**Authors:** Jenny M Groarke, Janice Richmond, Mary Grace Kelly, Jenny McSharry, AnnMarie Groarke, Tommy Kerr, Nina Singaroyan, Owen Harney, Charlene Haughey, Liam Glynn, Eimear Masterson, Aoife O Donnell, Karen Duffy, Jane Walsh

**Affiliations:** 1 School of Psychology, Queen's University Belfast, Belfast, UK Belfast United Kingdom; 2 Letterkenny University Hospital Donegal Ireland; 3 School of Psychology, National University of Ireland, Galway Galway Ireland; 4 Health Research Institute and Graduate Entry Medical School, University of Limerick Limerick Ireland

**Keywords:** mHealth, SMS, activity tracker, behavior change technique, health behavior change, obesity, cancer

## Abstract

**Background:**

Cancer survivorship in Ireland is increasing in both frequency and longevity. However, a significant proportion of cancer survivors are overweight. This has negative implications for long-term health outcomes, including increased risk of subsequent and secondary cancers. There is a need to identify interventions, which can improve physical and psychological outcomes that are practical in modern oncology care. Mobile health (mHealth) interventions demonstrate potential for positive health behavior change, but there is little evidence for the efficacy of mobile technology to improve health outcomes in cancer survivors.

**Objective:**

This study aims to investigate whether a personalized mHealth self-management lifestyle program is acceptable to participants and can improve physical and psychological outcomes of a subgroup of cancer survivors with increased health risks related to lifestyle behaviors.

**Methods:**

A sample of 123 cancer survivors (body mass index >25 kg/m^2^) was randomly assigned to the control (n=61) or intervention (n=62) group. The intervention group attended a 4-hour tailored lifestyle information session with a physiotherapist, dietician, and clinical psychologist to support self-management of health behavior. Over the following 12 weeks, participants engaged in personalized goal setting to incrementally increase physical activity (with feedback and review of goals through short message service text messaging contact). Objective measures of health behavior (ie, physical activity) were collected using Fitbit (Fitbit, Inc). Data on anthropometric, physiological, dietary behavior, and psychological measures were collected at baseline (T0), 12 weeks (T1; intervention end), and 24 weeks (T2; follow-up). Semistructured interviews were conducted to explore the retrospective acceptability of the Moving On program from the perspective of the recipients.

**Results:**

This paper details the protocol for the Moving On study. The project was funded in August 2017. Enrolment started in December 2017. Data collection completed in September 2018. Data analysis is underway, and results are expected in winter 2019.

**Conclusions:**

The results of this study will determine the efficacy and acceptability of an mHealth intervention using behavior change techniques to promote health behaviors that support physical health and well-being in cancer survivors and will therefore have implications for health care providers, patients, health psychologists, and technologists.

**International Registered Report Identifier (IRRID):**

DERR1-10.2196/13214

## Introduction

### Background

In Ireland, an average of 37,000 new cases of cancer are diagnosed each year, and it is predicted that the incidence of cancer will double by 2040 [1]. At the same time, cancer survivorship in Ireland is increasing, with survival at 5 years from diagnosis having increased to 62% overall [1].

There is consistent evidence of a positive association between being overweight or obese and all-cause morbidity and mortality [2]. High body mass index (BMI), poor diet, and lack of physical exercise are identifiable risk factors for cancer development, and in cancer survivors, these factors can increase the risk of a secondary cancer or a subsequent primary cancer [3,4]. Cancer and cancer treatment can result in physical inactivity and loss of muscular strength [5]. Previous research has identified that approximately 50% of cancer survivors are overweight [6], and research in women has linked obesity to a 46% increased risk for eventual development of distant metastases [7]. Cognizant of the consequences of morbidity and mortality, there is a need to facilitate rehabilitation of cancer survivors to reduce BMI and improve physical and psychological health.

Mobile health (mHealth) is the practice of medicine and public health supported by mobile devices (eg, mobile phones, smartphones, tablets, mobile apps, and wearable monitors). The use of mobile apps has been associated with significant reductions in weight and BMI [8]. Mobile technology has also been shown to assist in behavior change for physical activity, and it offers much potential for sustainable lifestyle change, as it provides feedback to consolidate self-management habits [9,10]. Therefore, mHealth tools may help meet the need to provide cost-effective behavior change interventions that support weight loss.

Although mHealth interventions hold significant potential, adopting a theory and evidence-based approach to intervention design is critical [11]. The Behavior Change Wheel (BCW) is a synthesis of 19 frameworks of behavior change [12]. The BCW, together with the Behavior Change Technique (BCT) Taxonomy, a standardized list of the active ingredients of behavior change interventions [13], enables researchers to develop and describe complex interventions in a systematic and rigorous way.

Systematic review evidence suggests that the use of relevant BCTs significantly increased the success of weight loss programs [14]. A systematic review of existing healthy eating and physical activity interventions identified the BCTs *self-monitoring* in combination with *goal setting* and *feedback* as the most effective [15]. A recent meta-analysis of 30 randomized controlled trials (RCTs) to increase physical activity among cancer survivors reported that certain BCTs (*prompts*, *social rewards*, and *graded tasks*) were associated with larger increases in physical activity. Interventions using a greater number of BCTs were associated with greater physical activity gains [16]. As such, these BCTs should be considered for inclusion in interventions aiming to increase physical activity.

Studies have found that both mHealth interventions and the inclusion of relevant BCTs can lead to positive health behavior change and weight loss and therefore, gains may be particularly great when mHealth and BCTs are combined. Digital interventions including a greater number of BCTs were found to have larger effects on health behavior change than interventions with fewer BCTs [17]. A review and meta-analysis of studies using activity monitors found that in people with obesity, physical activity increases were greatest when the BCTs *goal setting* and *feedback* were incorporated in the mHealth intervention [18]. A systematic content analysis of the BCTs provided by wearable activity monitors concluded that most monitors included *self-monitoring*, *goal setting*, and *feedback* [19]. More generally, the review by Michie et al [15] found these to be the most effective BCTs for promoting healthy diet and physical activity.

mHealth interventions incorporating relevant BCTs have the potential to support weight loss [16,17,19]. However, there are a limited number of mHealth interventions using BCTs with cancer survivors. In the previously mentioned meta-analysis of 30 physical activity RCTs for cancer survivors [16], only 2 studies [20,21] used digital technologies as the mode of delivery (MOD) for BCTs to increase physical activity. The study by Bantum et al [20] found that a 6-week Web-based self-management workshop increased self-reported strenuous physical activity of cancer survivors. The other study with breast cancer survivors used *prompts* delivered by email in declining frequency over 12 weeks and reported significant group differences in self-reported physical activity levels postintervention [21]. These studies highlight that there is potential for digital health interventions to improve lifestyle behaviors among cancer survivors. Yet, more evidence is needed regarding the effectiveness of interventions using mobile technologies with cancer survivors on objective health outcomes.

### Aims and Objectives

The aim of this multidisciplinary research study is to investigate whether or not a personalized mHealth (mobile technology) self-management lifestyle program can improve physical and psychological outcomes of a subgroup of cancer survivors with increased health risks related to lifestyle behaviors. More specifically, this project will examine the impact of lifestyle advice and personalized goal setting compared with standard medical care on both clinical and psychological outcomes. Furthermore, this study will explore the acceptability of this intervention to participants receiving the Moving On program.

## Methods

### Study Design

A 2-arm, parallel, open-label RCT design was used to investigate the impact of a personalized mHealth intervention versus standard care on primary and secondary health outcomes. Eligible participants were randomized to either the intervention or the standard care control condition using a computerized random number generator. Assessments took place before randomization (T0; baseline), at 12 weeks (T1; intervention end), and at 24 weeks (T2; follow-up). The study was not blinded.

On completion of the study, a series of semistructured interviews were carried out to assess retrospective acceptability of the intervention from the perspective of the recipients. Purposeful maximum variation sampling was used. The theoretical framework of acceptability of health care interventions was used as a topic guide [22]. Specifically, open-ended questions were asked regarding participant’s affective attitude toward the intervention, the intervention’s coherence, participant burden, perceived effectiveness, and participants’ sense of self-efficacy.

### Study Setting

Recruitment and assessments took place in Letterkenny University Hospital, Co. Donegal, Ireland.

### Ethics Approval

The design of this study was approved by the National University of Ireland, Galway Research Ethics Committee on September 12, 2017 (Ref: 17/MAY/20), and by the Research Ethics Committee at Letterkenny University Hospital on May 2, 2017.

### Intervention

#### Development

**Figure 1:**
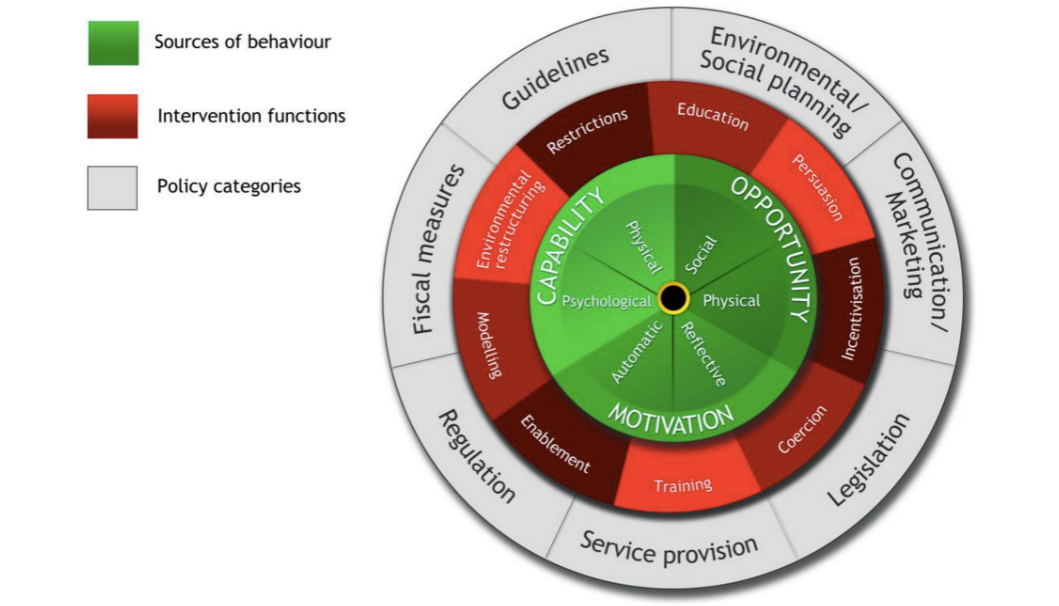
The behavior change wheel reproduced with permission from Michie et al.

The Moving On program is an intervention using BCTs and mobile technology to promote self-management of lifestyle behavior among cancer survivors who are overweight. The intervention was designed following the BCW [12] presented in Figure 1. The COM-B system at the center of the wheel proposes that people need the capability (C), opportunity (O), and the motivation (M) to perform a behavior (B). The second and third layers of the wheel describe intervention functions, the broad categories of means by which an intervention can change behavior, and policy categories that can support behavior change. The final step in intervention design is to identify BCTs and modes of delivery likely to be effective based on previous research.

Two behavioral targets were identified to improve health and well-being outcomes, which were to increase physical activity and improve diet. The functions of the first component of the intervention are *education* and *training*. The lifestyle information and education session aims to increase participants’ psychological capability to change behavior by imparting knowledge and skills to increase healthy eating and exercise. The function of the second intervention component (goal setting) was *enablement*. Participants were provided with a Fitbit activity tracker influencing their opportunity to increase physical activity. Participants also received short message service (SMS) text messaging contact from the behavioral science researcher regarding physical activity goals aiming to influence their motivation to increase exercise.

Next, a detailed intervention plan was developed and described using the BCT Taxonomy version 1 [13]. Key BCTs were selected on the basis of successful application in previous interventions targeting physical activity and dietary behavior change [14-16,18]. The full intervention specification detailing the content (BCTs) and MOD [23] for each BCT are described for both components of the intervention in Tables 1 and 2. The lifestyle information and education session was delivered primarily through face-to-face human contact in real time to groups of participants. In contrast, the goal setting intervention was delivered through human contact at a distance using nonautomated SMS text messages and facilitated using digital wearable technology.

**Table 1:** Description of intervention content of the lifestyle information and education session (week 1) specified in terms of the Behavior Change Technique Taxonomy Version 1 and the Mode of Delivery Taxonomy.

BCT^a^ and definition	Application in this study
Goal setting (outcome): set or agree on a goal defined in terms of a positive outcome of wanted behavior.	All participants enrolled in the research study are encouraged to lose weight by increasing their level of physical activity and improving their diet; MOD^b^: human and face-to-face.
Provide information on consequences of behavior to the individual: provide information (eg, written, verbal, and visual) about health consequences of performing the behavior.	Participants are given information on physical activity and healthy eating tailored to cancer survivors. For example, that high-impact activity, such as walking, is safe for cancer survivors.
Demonstration of the behavior: provide an observable sample of the performance of the behavior.	A series of physical exercises are demonstrated by the physiotherapist, and participants are shown how to use body parts (eg, hand) as a visual guide for healthy portion size. Information is summarized in a written information sheet to take home; MOD: human and face-to-face; MOD: printed material and leaflet
Provide instruction on how to perform the behavior: advise or agree on how to perform the behavior.	A series of physical exercises are demonstrated by the physiotherapist, and participants are shown how to use body parts (eg, hand) as a visual guide for healthy portion size. Information is summarized in a written information sheet to take home; MOD: human and face-to-face; MOD: printed material and leaflet
Problem solving: analyze, or prompt the person to analyze, factors influencing the behavior and generate or select strategies that include overcoming barriers and/or increasing facilitators.	Using a worksheet, each participant is prompted to record their goal, identify barriers to their goal, define the barrier in terms of it being a personal, environmental, social, or organizational barrier, and finally to identify strategies to overcome each barrier identified; MOD: human and face-to-face
Goal setting (behavior): set or agree on a goal defined in terms of the behavior to be achieved	Participants agree to gradually increase their physical activity level, including their average daily step count
Action planning: prompt detailed planning of performance of the behavior, must include at least 1 of context, frequency, duration, and intensity	Each participant is prompted to make a plan to increase their physical activity level toward the recommended 10,000 steps per day and perform the exercises recommended by the physiotherapist at a time/place of their choosing from a choice of schedules; MOD: human and face-to-face

^a^BCT: behavior change technique.

^b^MOD: mode of delivery.

**Table 2:** Description of intervention content of the goal setting intervention (weeks 4-12) specified in terms of the Behavior Change Technique Taxonomy Version 1 and the Mode of Delivery Taxonomy.

BCT^a^ and definition	Application in this study
Self-monitoring of behavior: establish a method for the person to monitor and record their behavior(s) as part of a behavior change strategy	Participant is provided with a Fitbit Alta activity tracker. Physical activity behavior is visually displayed on the screen, and a log of their previous activity is recorded and displayed on the app interface; MOD^b^: digital, wearable, and accessory; MOD: digital, phone, and app
Feedback on behavior: monitor and provide informative or evaluative feedback on performance of the behavior and must include one of form, frequency, duration, and intensity.	Once a week, the participant is contacted by SMS^c^ text messages to inform them of their average daily step count; MOD: human, distance, and SMS text message
Goal setting (behavior): set or agree on a goal defined in terms of the behavior to be achieved	Participant is contacted by SMS text messages with a daily step count goal for the following week; MOD: human, distance, and SMS text message
Graded tasks: set easy-to-perform tasks, making them increasingly difficult, but achievable, until behavior is performed	The participants’ step count goal is calculated by adding 10% to their previous week’s average daily step count and sent by SMS text messages; MOD: human, distance, and SMS text message
Social reward: arrange verbal or nonverbal reward if and only if there has been effort and/or progress in performing the behavior (includes *positive reinforcement*)	The participant receives a congratulatory SMS text message if they successfully achieve their step count goal that week; MOD: human, distance, and SMS text message
Review behavior goal(s): review behavior goal(s) jointly with the person and consider modifying goal(s) or behavior change strategy in light of achievement	If the participant does not successfully achieve their step count goal, a new goal is calculated based on their previous week’s activity level and sent by SMS text messages; MOD: human, distance, and SMS text message

^a^BCT: behavior change technique.

^b^MOD: mode of delivery.

^c^SMS: short message service.

#### Description

The intervention had 2 components: (1) a lifestyle information and education session delivered by health professionals (specifically, 3 physiotherapists, 1 dietician, and 1 clinical psychologist) at Letterkenny University Hospital and (2) goal setting intervention delivered by a behavioral science researcher using mobile technology.

##### Lifestyle Information and Educational Session (Week 1)

Participants in the intervention group attended a 1-day (4-hour) session in small groups of 10 to 15 people, where they received personalized and tailored lifestyle information from physiotherapists, a dietician, a clinical psychologist, and a behavioral scientist. Participants received a comprehensive presentation from each specialist. The physiotherapists demonstrated a series of daily strengthening exercises that were suitable and safe posttreatment. Moderate physical activity for 30 min 6 days a week, 45 min 4 days a week, or 10 min 2 times a day was recommended. Participants were advised to choose a personal activity schedule that best fit their lifestyle. Their preference or adherence to a particular schedule was not measured or controlled. The behavioral science researcher prescribed a weekly increase of 10% in average daily step count over the course of the program. The dietician advised participants to reduce their calorific intake, reduce red meat, processed meat, salt and sugar, and increase fruit, vegetable, and fiber intake. Participants were provided with an information sheet to take home summarizing the key messages from the lifestyle information and education session and booklets by the World Cancer Research Fund on healthy eating and physical activity. The BCTs included in this session are shown in Table 1.

##### Goal Setting Intervention (Weeks 4-12)

Participants in the intervention group self-monitored their physical activity using their Fitbit. In addition, participants in the intervention group were contacted by the behavior specialist through SMS text messages on weeks 4 to 11 to provide feedback on their average daily step count, review physical activity goals, and set graded tasks (increase daily step count by 10%). Participants gradually increased their physical activity (+10% each week) toward the recommended 10,000 steps per day. Participants continued to self-monitor their progress for the remaining 3 months of the study without review/feedback from the behavior specialist. The BCTs included in the goal setting intervention are presented in Table 2.

##### Materials

Each participant was provided with a Fitbit activity tracker and registered with a Fitbit user account. The Fitbit is an accelerometer-based device that is worn on the wrist. The intervention group wore the Fitbit Alta to track health outcomes. Summary data (eg, step count and active minutes) were visible on the device display, and additional data (eg, sleep data) were available on the Fitbit app dashboard.

#### Control Condition

Although the intervention was specifically designed to deliver key BCTs, a number of BCTs are present in standard medical care and therefore also present in the control condition in this study (summarized in Table 3).

**Table 3:** Description of control condition content specified in terms of the Behavior Change Technique Taxonomy Version 1 and the Mode of Delivery Taxonomy*.*

BCT^a^ and definition	Application in this study
Goal setting (outcome): The person is encouraged to set a general goal that can be achieved by behavioral means but is not defined in terms of behavior (eg, to lose weight), as opposed to a goal based on changing behavior.	All participants enrolled in the research study are encouraged to lose weight by increasing their level of physical activity and improving their diet; MOD^b^: digital, wearable, and accessory
Provide information on consequences of behavior to the individual: information about the benefits and costs of action or inaction to the individual or tailored to a relevant group based on that individual’s characteristics.	Participants in the control group attend a small group session to receive their Fitbit Flex 2. Standard advice regarding healthy diet and lifestyle is provided at this session; MOD: printed material and leaflets
Self-monitoring of behavior: establish a method for the person to monitor and record their behavior(s) as part of a behavior change strategy.	Participant is provided with a Fitbit Flex 2 activity tracker. The visual display does not provide summary data, the app interface is modified to not present summary data, and the participant is not given any method for monitoring/recording their activity level using Fitbit; MOD: digital, wearable, and accessory

^a^BCT: behavior change technique.

^b^MOD: mode of delivery.

##### Materials

All participants in the control group were provided with Fitbit Flex 2 to track health outcomes. The display panel on this device does not present summary data (ie, step count and number of active minutes), and the application dashboard was modified to not display summary data.

On being recruited to the study based on meeting the eligibility criteria (BMI ≥25 kg/m^2^), all participants were encouraged to lose weight (ie, BCT; *goal setting [outcome]*) because of health care professionals’ duty of care. Participants in the control group attended a 15-min session in small groups of 10 to 15 participants, where they received a Fitbit Flex 2. In addition, as in standard medical care, there was provision of health information at this session (ie, BCT *information on consequences of behavior to the individual*, but not BCT *instruction on how to perform the behavior* or *demonstration of the behavior*). Therefore, leaflets were made available, and standard advice was available from oncology nursing staff on request. No further information was given at this time. Finally, participants in the control group wore the Fitbit Flex 2 for monitoring their physical activity, but without *goal setting (behavior)*. The visual display on the device and the app limited but did not eliminate their ability to *self-monitor* behavior.

### Participants

#### Inclusion Criteria

Adults aged 18 to 70 years, having a calculated BMI equal to or greater than 25 kg/m^2^, with a solid cancer and who had completed cancer treatment (except for hormone therapy), attended Oncology Services in Letterkenny University Hospital during the recruitment phase, and had a willingness to use mobile technology were eligible to participate. A total of 10 eligible participants who did not own a smartphone were provided with an Amazon Fire 7 Tablet.

#### Recruitment and Consent

The clinical team identified 159 eligible participants who were identified sequentially from the oncology outpatient waiting list. The research team contacted them by telephone and described the aims and design of the study. Prospective participants who were interested in the study were sent an invitation letter, participant information sheet, and consent form. Informed written consent was provided by 123 participants (77.3% response rate) who attended baseline assessments. Participant characteristics are described in Table 4.

**Figure 2:**
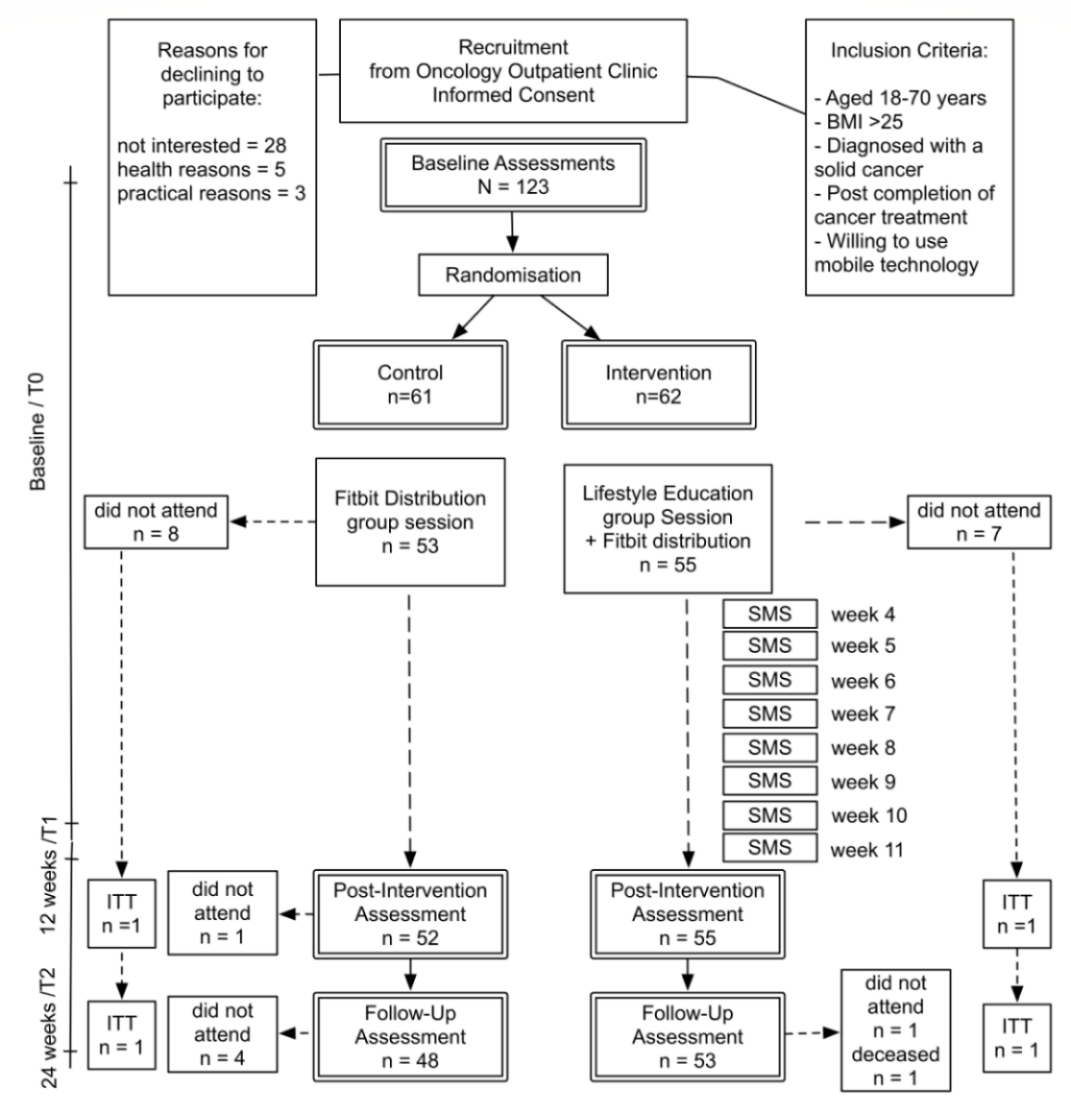
Flow of participants through each stage of the current randomized controlled trial. BMI: body mass index; ITT: intention-to-treat; SMS: short message service.

Of the 36 participants who did not consent to participate, 28 were not interested, 3 were waiting for surgery, 1 had chronic obstructive pulmonary disease, 1 was undergoing recurrence workup, 2 had young children, and 1 did not drive (see Figure 2).

**Table 4:** Participants’ characteristics at baseline assessment.

Characteristics		Control	Intervention	2-tailed *t* test (*df*)	*P* value
Age, mean (SD)		59.24 (7.65)	55.61 (8.05)	2.39 (105)	.02
Weight (kg), mean (SD)		87.10 (16.32)	84.18 (13.98)	0.99 (105)	.32
BMI^a^ (kg/m^2^), mean (SD)		32.64 (5.41)	30.33 (3.99)	2.53 (105)	.01
Gender, female:male		49:4	42:12	—^b^	—
**Have you ever been told by a doctor that you have or have had any of the following conditions?, n (%)**					
	Angina	1 (1.9)	2 (3.7)	—	—
	Heart attack	3 (5.7)	1 (1.9)	—	—
	High blood pressure	19 (35.8)	18 (33.3)	—	—
	Stroke	3 (5.7)	1 (1.9)	—	—
	Diabetes	5 (9.4)	6 (11.1)	—	—
	High cholesterol	21 (39.6)	20 (37.0)	—	—
	Depression	12 (22.6)	9 (16.7)	—	—
	Anxiety	12 (22.6)	12 (22.2)	—	—

^a^BMI: body mass index.

^b^Not applicable.

#### Sample Size

The statistical program G*Power was used to conduct power analysis. With 2 groups (intervention and control), 3 measurements (baseline, time 1, and time 2), an assumed correlation among repeated measures of 0.3, and a small-medium effect size and a power of 0.8, the recommended sample size for repeated measures analysis of variance (ANOVA) was 102. Sample size calculations were made considering attrition rates (approximately 20%) observed in similar studies using mobile technology interventions with cancer survivors [24].

### Procedure

A flow diagram of the progress through each phase of this 2-group parallel randomized trial is presented in Figure 2. A total of 123 eligible participants attended baseline assessments. Participants were randomized to the control or intervention arm. Of 123 participants, 62 assigned to the intervention group were invited to attend a lifestyle information and education session where they would also receive their Fitbit activity monitor, and 55 were able to attend. During the lifestyle information and education session, each participant was provided with a Fitbit Alta. The Fitbit activity tracker was set up and paired with the participants’ mobile device. The participant was given an information sheet with instructions on how to synchronize their Fitbit device and app and asked to perform this weekly.

The remaining 61 participants assigned to the control group were invited to an appointment where they would be provided with a Fitbit activity monitor, and 53 were able to attend. During this meeting, each participant was provided with a Fitbit Flex 2. The Fitbit was paired with the participants’ mobile device. The participant was given an information sheet with instructions on how to synchronize their Fitbit and asked to perform this weekly or at least once a month to prevent loss of data.

All participants wore a Fitbit® activity monitor for the 6-month duration of the study. Participants in the intervention group received weekly personalized SMS text messages, including feedback on their physical activity level and a personalized physical activity goal each week for 8 weeks.

All participants were invited to a postintervention assessment (12 weeks later) to determine efficacy of the lifestyle information and education session and personalized goal setting mHealth intervention for increasing physical activity and improving clinical and psychological outcomes.

All participants continued to wear Fitbit for the remainder of the study, but the personalized goal setting intervention had ceased after 12 weeks. To determine if any effects of a lifestyle information and education session and personalized goal setting mHealth intervention were maintained 3 months later, all participants were invited to a follow-up assessment (6 months after baseline assessment).

At the conclusion of the study, all participants in the intervention group were invited to be interviewed about their experience of the Moving On program, and 13 interviews were conducted.

### Outcomes

The outcomes measured at baseline (T0), 12 weeks (T1; intervention ends), and 24 weeks (T2; follow-up) are described below.

#### Clinical Outcomes

##### Anthropometric Measurements

Weight was measured in light clothing without shoes in kilogram plus 2 decimal places using a calibrated Seca scale. Height was recorded without shoes in centimeters plus 2 decimal places using a stadiometer. BMI was calculated using weight and height. Waist circumference was measured at the halfway point between the hip bone and lowest rib using a stretch-resistant measuring tape [25]. To standardize measurement, one set of scales and stadiometer were used on all participants at each assessment.

##### Functional Exercise Capacity

The 6-min walk test is a clinical exercise test that measures the distance walked in 6 min on a hard, flat surface. Systolic blood pressure, diastolic blood pressure, heart rate, blood oxygen saturation, subjective fatigue, and dyspnea were measured pretest, posttest, and 4 min later.

#### Psychological Outcomes

Medical Outcomes Survey Short Form (RAND36) [26] is composed of 36 items, measuring 8 individual subscales that represent 3 general areas of health-related quality of life: physical, emotional, and social well-being. The subscales include physical functioning, role function–physical (role limitations caused by physical factors), role function–emotional (role limitations caused by emotional factors), bodily pain, social functioning, emotional well-being, energy/fatigue, and perceived general health. Each subscale is standardized on a scale from 0 to 100, with higher scores indicating better functioning.

The Three-Item Loneliness Scale [27] consists of 3 questions such as “How often do you feel that you lack companionship?” The responses are coded 1: hardly ever, 2: some of the time, and 3: often. Scores range from 3 to 9, with higher scores indicating greater loneliness.

The Brief Fatigue Inventory [28] comprises 9 items measured on a 10-point Likert scale. The scale is composed of 2 subfactors that assess the severity of fatigue and its effects on the respondent’s ability to perform activities of daily living. Scores range from 0 to 90. Higher scores represent worse self-reported fatigue.

The General Self-Efficacy Scale [29] is a 10-item measure with answers ranging from *not true at all* to *exactly true*. It assesses the participants’ belief in their ability to succeed in certain situations. Scores range from 10 to 40, with higher scores indicating higher self-efficacy.

Exercise self-efficacy was assessed using a 4-item measure developed in a previous study by Armitage [30]. Items were as follows: “To what extent do you see yourself as being capable of participating in regular physical activity? incapable–capable”; “How confident are you that you will be able to participate in regular physical activity? not very confident–very confident”; “I believe I have the ability to participate in regular physical activity. definitely do not–definitely do”; and “How much personal control do you feel you have over participating in regular physical activity? no control–complete control. Items were measured using 7-point scales.

Social support for physical activity was measured using a 3-item measure developed by Molloy et al [31] that was based on an earlier measure [32]. The items began with the stem “In the last week I...had somebody to encourage me to participate in physical activity on a regular basis,...had somebody to participate in physical activity with me,...felt supported in having a regular pattern of physical activity.” The responses to the 3 items were on a 7-point scale and ranged from 1: disagree to 7: agree.

#### Health Behavior Outcomes

Self-reported physical activity level was measured using the 4-item Godin Leisure-Time Exercise Questionnaire [33]. Respondents rate the frequency of 15-min bouts of strenuous, moderate, and mild exercise in a 7-day period. Participants also rate how often they engage in regular activity sufficient to break a sweat (1 often, 2 sometimes, and 3 never). Higher scores indicate higher subjective physical activity.

Objective physical activity level (ie, average daily step count) was measured continuously using the Fitbit activity tracker.

Dietary data were collected using the European Prospective Investigation into the Cancer and Nutrition (EPIC) Norfolk Food Frequency Questionnaire (FFQ) [34]. Participants were asked to report the frequency of 130 different foods and beverages consumed over the previous 3 months. The FFQ EPIC Tool for Analysis [35] provides estimates of 10 food groups.

#### Acceptability

A 5-item acceptability measure was created based on the topic guide for the semistructured interviews. The topic guide itself was informed by the theoretical framework of acceptability [22]. Using a 5-point Likert scale, participants in the intervention were asked to rate their satisfaction with the intervention (affective attitude), perceived effectiveness of the intervention, their confidence in performing the behaviors required to participate (self-efficacy), the perceived amount of effort required to participate (burden), and the extent to which they understand how the intervention is intended to work (coherence).

### Statistical Analysis

To maximize power and conform to intention-to-treat analysis, missing data will be handled using multiple imputation methods (ie, the expectation-maximization algorithm) if assumptions regarding mechanisms of missingness are met.

A series of 3 (baseline, T1, and T2) × 2 (control and intervention) mixed ANOVAs will be performed to determine the efficacy of a lifestyle information and education session and goal setting mHealth intervention on clinical, psychological, and health behavior measures.

Independent sample *t*-tests will be used to analyze group differences (control and intervention) in average daily step count across the 24 weeks of the study.

Thematic analysis of interview transcripts will be used to explore the acceptability of the Moving On program for recipients.

Quantitative data will be analyzed with IBM SPSS Statistics 24. NVivo 12 will be used to facilitate organization and analysis of qualitative data.

## Results

The recruitment for this study commenced in December 2017 and data collection began in January 2018. Data collection was completed by September 2018, and analysis is underway. Results are expected to be submitted for publication in winter 2019.

## Discussion

This protocol describes an RCT designed to evaluate the efficacy of an intervention using mobile technology and BCTs to improve health and well-being outcomes in a sample of cancer survivors with a BMI of 25 kg/m^2^ or greater. Strengths of the study protocol include a description of the intervention content in terms of a standardized list of BCTs [13] and the MOD taxonomy [23], as well as a description of control condition content using the same standardized descriptors. Qualitative elements examining the acceptability of the intervention are an additional strength of the study.

Cancer survivors require additional support to self-manage lifestyle behaviors. mHealth technology may provide a cost-effective solution within modern oncology care. However, there is limited evidence for the effectiveness of mHealth interventions for behavior change with cancer survivors. mHealth is a novel area of research, and although it holds enormous potential for improved health care delivery in the future, it currently lacks a strong evidence base [36]. This study evaluating the efficacy of an mHealth intervention using evidence-based BCTs to improve health and well-being outcomes in a sample of cancer survivors who are overweight represents an important contribution to the field. If results of the study find the *Moving On* program to be effective and acceptable to participants, possibilities for full-scale national roll-out will be explored.

The findings of this study will be of interest to health care professionals and patients, health psychologists with an interest in behavior change, and those developing new technologies to support health behavior change.

## Abbreviations

ANOVA: analysis of variance
BCT: behavior change technique
BCW: behavior change wheel
BMI: body mass index
EPIC: European Prospective Investigation into the Cancer and Nutrition
FFQ: Food Frequency Questionnaire
mHealth: mobile health
MOD: mode of delivery
RCT: randomized controlled trial
SMS: short message service
